# Correction: Doum, D., et al. Dengue Seroprevalence and Seroconversion in Urban and Rural Populations in Northeastern Thailand and Southern Laos. *Int. J. Environ. Res. Public Health* 2020, *17*, 9134

**DOI:** 10.3390/ijerph18041439

**Published:** 2021-02-04

**Authors:** Dyna Doum, Hans J. Overgaard, Mayfong Mayxay, Sutas Suttiprapa, Prasert Saichua, Tipaya Ekalaksananan, Panwad Tongchai, Md. Siddikur Rahman, Ubydul Haque, Sysavanh Phommachanh, Tiengkham Pongvongsa, Joacim Rocklöv, Richard Paul, Chamsai Pientong

**Affiliations:** 1 Tropical Medicine Graduate Program, Academic Affairs, Faculty of Medicine, Khon Kaen University, Khon Kaen 40002, Thailand; dynadoum@gmail.com (D.D.); sutasu@kku.ac.th (S.S.); prasertsai@kku.ac.th (P.S.); 2 Department of Microbiology, Faculty of Medicine, Khon Kaen University, Khon Kaen 40002, Thailand; hans.overgaard@nmbu.no (H.J.O.); tipeka@kku.ac.th (T.E.); panwad1622@gmail.com (P.T.); siddikur@brur.ac.bd (M.S.R.); 3 Faculty of Science and Technology, Norwegian University of Life Sciences, P.O. Box 5003, 1432 Ås, Norway; 4 Institute of Research and Education Development (IRED), University of Health Sciences, Ministry of Health, P.O. Box 7444, Vientiane 43130, Laos; mayfong@tropmedres.ac (M.M.); sysavanhp@gmail.com (S.P.); 5 Lao-Oxford-Mahosot Hospital-Wellcome Trust Research Unit (LOMWRU), Mahosot Hospital, Vientiane 43130, Laos; 6 Centre for Tropical Medicine and Global Health, Nuffield Department of Clinical Medicine, Old Road Campus, University of Oxford, Oxford OX3 7LG, UK; 7 HPV & EBV and Carcinogenesis Research Group, Khon Kaen University, Khon Kaen 40002, Thailand; 8 Department of Statistics, Begum Rokeya University, Rangpur 5400, Bangladesh; 9 Department of Biostatistics and Epidemiology, University of North Texas Health Science Center, Fort Worth, TX 76177, USA; mdubydul.haque@unthsc.edu; 10 Savannakhet Provincial Health Department, Savannakhet 13000, Laos; tiengkhampvs@gmail.com; 11 Department of Public Health and Clinical Medicine, Umeå University, 90187 Umeå, Sweden; joacim.rocklov@umu.se; 12 Institut Pasteur, Unité de la Génétique Fonctionnelle des Maladies Infectieuses, CNRS UMR 2000, 75015 Paris, France

## Text Correction

There was an error in the original article [1]: **ethylenediamine tetraacetic acid (EDTA)-tubes** was stated incorrectly.

A correction has been made **to 2. Materials and Methods, 2.2. Blood Sample Collection, Line 120, Paragraph 6:**

**VACUETTE clot activator tubes**

The authors apologize for any inconvenience caused and state that the scientific conclusions are unaffected. The original article has been updated.
